# Complete Genome Sequence of a Novel Human Papillomavirus Isolated from Oral Rinse

**DOI:** 10.1128/genomeA.01281-17

**Published:** 2017-11-09

**Authors:** Zigui Chen, Martin C. S. Wong, Po Yee Wong, Wendy C. S. Ho, Miaoyin Liang, Apple C. M. Yeung, Paul K. S. Chan

**Affiliations:** a Department of Microbiology, The Chinese University of Hong Kong, Hong Kong SAR, China; b Jockey Club School of Public Health and Primary Care, The Chinese University of Hong Kong, Hong Kong SAR, China; c State Key Laboratory of Digestive Disease, Institute of Digestive Disease, The Chinese University of Hong Kong, Hong Kong SAR, China; d Stanley Ho Center for Emerging Infectious Diseases, Faculty of Medicine, The Chinese University of Hong Kong, Hong Kong SAR, China

## Abstract

A novel human papillomavirus (HPV TG550) isolated from the oral rinse of a Chinese male resident was fully characterized. The L1 open reading frame of HPV TG550 shares 82.5% nucleotide sequence similarity with its closest relative, HPV166, and clusters within the species group *Gammapapillomavirus 19*.

## GENOME ANNOUNCEMENT

Human papillomaviruses (HPVs) are a heterogeneous group of circular double-stranded DNA viruses with a size around 8 kb and infect the epithelial surfaces of genital and cutaneous sites of humans. A distinct HPV type is defined when the complete L1 open reading frame (ORF) is less than 90% similar to those of the characterized types (1, 2). Currently, nearly 200 types of human papillomaviruses have been fully characterized and are classified mainly in 5 genera, *Alphapapillomavirus*, *Betapapillomavirus*, *Gammapapillomavirus*, *Mupapillomavirus*, and *Nupapillomavirus*, within the family *Papillomaviridae*. The *Alpha-PV* genus contains all of the genital oncogenic HPV types associated with cancers (3, 4).

In this study, we characterized the complete genome of a novel HPV type isolated from the oral rinse of a 22-year-old Chinese male resident without visible oral lesions. Viral DNA was initially detected using a multiplexed next-generation sequencing (NGS) assay targeting a 125-bp fragment within the L1 ORF (5). A BLASTn search of the sequence against an HPV database showed less than 90% similarity to characterized PV types. The total DNA was further enriched using rolling-circle amplification (RCA) with random primers, and we conducted metagenomic sequencing on an Illumina HiSeq4000 instrument at Weill Cornell Medicine Genomics Resources Core Facility, New York, using paired-end reads. The short reads were filtered for human genome contamination and *de novo* assembled; a total of 11 valid contigs (243 to 1,420 bp) covered nearly 80% of the complete genome. Type-specific primers were designed based on NGS contigs to amplify the complete genome in three overlapping fragments. The complete genome sequence was assembled and validated using a proofreading polymerase, followed by Sanger sequencing using a primer-walking strategy.

This novel HPV genome (HPV TG550) is 7,216 bp in size, with a GC content of 37.4%. It contains five early genes (E6, E7, E1, E2, and E4) and two late genes (L1 and L2). The complete L1 ORF shows 82.5% nucleotide sequence similarity with its closest relative, HPV 166 (HPV166, GenBank accession number JX413104), meeting the criteria to be considered a distinct HPV type. Phylogenetic trees using either the L1 ORF or the complete genome sequence unambiguously cluster HPV TG550 into the species group *Gammapapillomavirus 19*, together with HPV161 (JX413109), HPV162 (JX413108), and HPV166. The nucleotide sequence of a small region of the HPV TG550 L1 ORF shares 97 to 99% similarities with three previously reported partial sequences, SIBX58 (KP768216; 360 bp), SE9 (JF906531; 235 bp), and SE71 (JQ250766; 222 bp).

The identification of novel HPVs reveals high genomic diversity of papillomaviruses. Further studies on the epidemiology, evolution, and pathogenesis of this novel virus are warranted.

### Accession number(s).

The complete genome sequence of HPV TG550 is available in GenBank under the accession number MF176072.
