# Erratum for Chen et al., “Complete Genome Sequence of a Novel Human Papillomavirus Isolated from Oral Rinse”

**DOI:** 10.1128/genomeA.00485-18

**Published:** 2018-05-31

**Authors:** Zigui Chen, Martin C. S. Wong, Po Yee Wong, Wendy C. S. Ho, Miaoyin Liang, Apple C. M. Yeung, Paul K. S. Chan

**Affiliations:** a Department of Microbiology, The Chinese University of Hong Kong, Hong Kong SAR, China; b Jockey Club School of Public Health and Primary Care, The Chinese University of Hong Kong, Hong Kong SAR, China; c State Key Laboratory of Digestive Disease, Institute of Digestive Disease, The Chinese University of Hong Kong, Hong Kong SAR, China; d Stanley Ho Center for Emerging Infectious Diseases, Faculty of Medicine, The Chinese University of Hong Kong, Hong Kong SAR, China

## ERRATUM

Volume 5, no. 45, e01281-17, 2017, https://doi.org/10.1128/genomeA.01281-17. Page 2, Acknowledgments, line 5: “ECS 2191114” should read “ECS 24100716.”

